# Inhalation of the Vapor of Sulphuric Ether for the Relief of Pain in Surgical Operations. Dr. Morton's Claims to Its Discovery

**Published:** 1847-10

**Authors:** 


### ARTICLE VIII.

### *Inhalation of the Vapor of Sulphuric Ether for the relief of Pain in Surgical Operations—Dr. Morton's Claims to its Discovery.*

Ever since the world was peopled, and man was endowed with a nervous organization; ever since pain was known to be painful, and human suffering was recognised as unpleasant, whatever would have a tendency to mitigate, or entirely subdue this "curse upon mankind," without producing a corresponding evil, has been a "consummation devoutly to be wished."

Philosophers have sought for it, but found it not. Philanthropists have yearned for its discovery with almost a religious zeal, and men of science have investigated in vain for the boon; nothing but some poor obscure foreshadowings of the mighty truth, nothing but a few blind intimations of the great desideratum have as yet been vouchsafed to man. Men have steeped their bodies in alcohol, until they were only arrested in the *experiment* by "spontaneous combustion!" They have soaked their systems in opium, and with its fumes have smoked their brains until they were literally tanned, and withered to a knot, in the vain hope of drinking of the waters of Lethe, without paying to nature the penalty of her broken laws. Still, in spite of this want of success, the march of discovery has been onward, and though ages have rolled over our heads and still found us ignorant, if we are to believe what is told us, it has been reserved for this latter day to be immortalized, in the discovery of the long hoped for *Letheon!* The cry of "land!" has been heard, and we are *told* the "new world" is before us! We are told that when sulphuric ether is inhaled into the lungs under peculiar circumstances, the whole nervous organization becomes insensible, even under the most severe surgical operations, and that after which, it recovers itself without suffering the least danger or inconvenience.

It is unnecessary for us, at this time, to speak of the manner in which this declaration has been received either on this or the other side of the Atlantic, nor does it become us to enter the field of combat to decide "who is the discoverer!"

Let it be sufficient for us as Editors of a Journal, devoted to the interests of a profession, for which the discovery in question promises especial and incalculable benefits, to inquire into the merits of the case, and see how far common prudence and common sense, will justify us in endorsing and recommending it to those who look to us with their confidence. Thus far we have deemed it prudent to say but little on the subject, a sense of justice, at least, would induce us to withhold passing judgment upon it hastily, or until a sufficient time had elapsed to have its character and influence fully known. Acting upon this principle, then, there can be now no longer reason for our withholding an article on the subject from our readers.

First, then, let us consider the article used, which is *sulphuric ether.* What is sulphuric ether? Sulphuric ether is obtained by distillation from a combination of sulphuric acid, alcohol and potassa. It is a colorless liquid, of a hot, pungent taste, has a strong affinity for oxygen, exceedingly volatile; when applied to the animal economy, and allowed to evaporate freely, it acts as a severe refrigerator, but when the evaporation is checked or protracted, it acts as a powerful stimulant—even producing resuscitation. Ether has ever been regarded as one of the most powerful, stimulating and dangerous narcotics in the whole materia medica. In its *administration,* the volatile gas arising from its evaporation is received into the lungs, and takes the place of the ordinary air we breathe.

Secondly. What would we naturally expect to be its influence on the human system under such circumstances? When received into the lungs, from its peculiar chemical properties we would expect it to cause burning and suffocating sensations from the elevated temperature produced by its evaporation in its confined situation. From its stimulating qualities, and accelerated or increased pulsation, either in rapidity or volume, together with an unusual distension of the blood vessels of the lungs, and, from our previous knowledge of its effect on the brain, we would naturally expect a similar influence there; at least, we should be justified in apprehending the worst results, especially, as death has been repeatedly produced by the inhalation of ether, long before its power to produce insensibility to pain was known.

Thirdly. What *is* its influence? Its immediate effects are, an increased circulation of blood, a burning sensation of the lungs, and, in many cases, decided symptoms of apoplexy and congestion of both the brain and lungs. It also produces insensibility to pain. Its reactive effects are, headache, vertigo, loss of memory, numbness, inflammation of the brain and lungs, spitting of blood, insanity and death.

Fourthly. *Why* does it produce these effects, or what is the *modus operandi?* When sulphuric ether is taken into the lungs, the blood in passing through them for oxydation, instead of coming in contact with pure atmospheric air, with its quantum of oxygen, is met with a smothering malaria, which not only bestows upon it its poisoning influences but absolutely robs it of all of its oxygen, or life-giving principle, besides effecting other chemical and deleterious influences to which we shall hereafter refer. Its heating and pungent character produces irritating and smothering sensations, which, together with its anodyne and stimulating qualities, produce an almost instantaneous and very increased circulation of the blood. So that, in every instance of its administration, it *produces congestion of the brain and lungs in a greater or less degree!* And this is the secret of the manner of its producing insensibility to pain. The action of the blood upon the brain is legitimately the same, as when in apoplexy or any other instance of congestion of the brain. The stentorious breathing, the quickened circulation, the determination of blood to the head, the death-like stupor, and the dilated pupils, are all symptoms which cannot be mistaken, of violent congestion and compression of the brain. Congestion of the brain and lungs must ever be produced on the inhalation of ether in a greater or less degree, and will ever produce its corresponding injurious effects according to the ability of the system to overcome its influence. Death has repeatedly been produced by the inhalation of sulphuric ether, and on dissection, the brain and lungs have been found *gorged with blood!* What does this prove, but that the blood-vessels of these two all important organs have been overstrained, even to bursting—that congestion has taken place. It is not strange that when the sensorium itself is completely deadened by compression, that all of its dependencies should be unfeeling and inactive.

Before entering into an exposition of its chemical effect upon the animal economy, we will consider another objection of a mechanical character. It is well known that during the administration of ether, the cause and effect follow closely upon each other; that its influence is almost instantaneous. The action upon the system is a violent one; from a state of quiet, the system is *suddenly* forced into one of dangerous and unnatural agitation. The pulse is either suddenly doubled—even tripled, or the volume of blood is fearfully increased. The labor of the lungs is suddenly increased to an unusual amount, the blood-vessels are suddenly distended to an unparalleled degree so suddenly, as in many instances, to cause their rupture. The compression produced upon the brain is so immediate and violent as almost to amount to a *shock!* and, in addition to this, the *subsidence* of the effect is almost as immediate. It requires but little reflection to convince us that the delicate organs and powers of the body must suffer and be endangered by this violence. Take a delicate piece of machinery, and suddenly bring it up to its highest degree of velocity, and you either greatly impair or ruin it; when at its greatest velocity, suddenly arrest it, and the consequences are the same. When we consider the striking analogy that exists in the two instances, are we not constrained to wonder that this "harp of a thousand strings should keep in tune" *at all*, under such influences. The wonder is not that death is the occasional consequence of its use, but that it does not occur oftener still.

In every instance wherein the human system suffers violence, and nature's laws are broken, the penalties of that violence must either directly or indirectly follow; and it matters not whether the human mind takes cognizance of the violence at the time or not, still the physical system must, as a penalty, suffer in a greater or less degree. How far, we may be unable to determine, but we can with no propriety declare that we have countermined this grand fundamental law of physical life. The fact, that the human system suffers pain, and violence and death, independent of all perceptible intelligence, comes up to us with an incontrovertible argument against the proposition. Our friends, laboring under the delirium of a fever, groan with the agony of a tortured and diseased organization, the pulse is quickened, the face is contorted, and the perspiration pours from the brow; and yet, when they are restored to intelligence, they have no recollection of pain; their emaciated frame and enfeebled condition, are all the evidences to them, of the storm that has passed over them. The subjects who inhale ether, often groan and writhe and act as though they were in the greatest agony, and yet, on being restored to consciousness, recollect nothing that has transpired. Unconsciousness can never be an invincible safeguard to the physical system under violence, it never has been so, and it never will. But let us consider this subject from another point of view.

"Dr. James H. Bickford has addressed to the London Morning Chronicle a solemn warning against the use of ether. He denies that the insensibility which it produces is no worse than that of drunkenness or asphyxia. There is a chemical alteration in the vital constituents of the blood; for not only is that deprived of its oxygen, and of the power of coagulation—like the black vitiated blood of malignant and putrid fever—but the corpuscules, whence fibrin are formed, are actually dissolved. Hence, the blood takes a long time to regain its life-supporting, flesh-forming character? wounds show everted edges and refuse to heal; and the patient often sinks into death. The use of ether also tends to produce tubercular consumption of the lungs; in thirty cases of death after the use of ether, in the Dublin Hospitals, the deaths could be traced to recent tubercles, believed to be the product of the ether."

Some have endeavored to obviate this deleterious chemical effect by having the patient breathe pure oxygen immediately after inhaling the ether, but as the objectionable result has *already* been produced, the oxygen can have but a comparative palliating influence.

A writer in the North British Review, after speaking of the danger of ether being used by those inclined to apoplexy, or those afflicted with pulmonary complaints or affections of the heart, of its injurious influence upon the brain, and of its causing convulsions, swoons and apoplectic attacks, says, "So far as we know, there is not one instance of fatal casualty which can be ascribed *directly* to the ether's use." This is somewhat singular when we have had such fearfully multiplied accounts of them. It is true, in most instances, the ether is said to be the *indirect* cause; but when effect follows so closely after cause, and in a line *so* direct, it is hardly honest to charge it with a deviation. But, unfortunately, we have many others of the most decided and direct character, and if we did not, it would make no difference in the premises. Notwithstanding the success of ether in Europe, we can hardly take up a foreign paper that does not record the death of a victim to its use.

The impossibility of graduating anything like a just relation between the quantity of ether to be given, and the amount of pain to be overcome, except in extreme cases, must ever be an objection to its indiscriminate use, and especially under trivial circumstances. If there be any graduation that can be relied upon, it must be that the danger diminishes in proportion to the severity of the operation, or the pain to be overcome. Yet the condition of the patient, the character of the operation, and a large variety of other circumstances must be taken into consideration, which can only be met by unusual skill and intelligent experience.

There is no propriety in our being told that very objectionable symptoms and fatal results do not often occur, that they do occur and cannot be anticipated is sufficient to condemn any thing like the general practice. The caution being sold at the same time with the right has hardly proven a safeguard against its effects, the principles upon which it acts upon the system are immutable, and must ever be in a greater or less degree the same, and consequently always *dangerous.*

If the administration of ether is ever justifiable, it must be under the most extraordinary circumstances, when an important surgical operation is to be performed, involving life and death. Into what utter insignificance then does the pain of the mere extraction of a tooth shrink, compared with the consequences which may occur; and in view of all the facts, are we not constrained to question the morality of those who will resort to it indiscriminately for that purpose.

The ether excitement has evidently had its day, and is destined soon to be superseded by that healthful reaction of common sense, which follows extremes in medical as well as other sciences. It will be laid aside as one of those powerful and dangerous agents, which we are justified in using only in the most extreme cases, that it may ultimately prove a blessing to mankind, we are not prepared to admit or deny; but that thus far it has produced a fearful amount of injury in comparison with the good, we have not the slightest doubt.

In regard to the array of distinguished names who advocate the drug, we have only to say: Truths as old as the establishment of medical science, common sense, and every exalted consideration, is against them; the better part of the profession is against them; and eventually, the whole scientific world must unite in condemning a course that is fraught with so much danger, and is liable to such fatal consequences.

In conclusion, then, we are constrained to record, that the use of sulphuric ether in the dental profession will be of small advantage, for the circumstances must rarely occur wherein its administratron would be justifiable.

That the administration of ether, or its effects, may ultimately be so modified as not to produce dangerous consequences, we with others most ardently hope, though we cannot see how it can be accomplished. Still, in any event, Dr. Morton is entitled to the respect and gratitude of the world at large, for his generous manifestations of an honest desire to promote the well being and alleviate the sufferings of mankind.

The above article was written previous to the reception of the communication from Dr. Morton, and at first sight we had thought of laying it aside, and penning another, more particularly applicable and appropriate as a *modifier* of Dr. M's. But on a "second sober thought" we have concluded, as Dr. Morton takes no particular issue with us, to publish it entire, adding merely a few subsequent reflections.

So far as regards the subject at issue—the propriety of using ether in the dental profession—we have no reason as yet to change materially our views as expressed in our article. We have heard that Dr. M. has abandoned his apparatus and recommends in its stead merely the use of a sponge. We certainly approve of this as far as it goes, for considerations which we have already indirectly expressed, and we are not at this time prepared to deny, that it may not lessen its liability to produce injury.

Dr. Morton has certainly most clearly and fairly proven to our minds, that he alone is the original discoverer; and whatever honor the future experience of the world may prove due to her benefactor in this respect, must in all justice be accredited to him. We have no misgivings in this respect, and trust as ardently as the doctor could wish, that his most sanguine hopes in regard to his discovery may be fully realised, and that well earned laurels may be the grateful and spontaneous acknowledgment due from a benefitted world.

We understand that Dr. M. has subjected himself to large sacrifices of time and pecuniary means in his endeavor to bring before the world a discovery which he hoped would so largely contribute to the happiness of mankind, for his honest and generous *intentions* at least, whether he is eventually successful or not, he is entitled to the respect and gratitude of all. But what we more particularly admire in the man, is the noble position which he has more recently taken. He has evidently considered his responsibilities to the world at large, and more particularly his indirect relation to a liberal association, devoted to the interests of humanity, and which must eventually exert a strong moral influence upon our profession. I allude to that spirit which actuated him in common with the fearless Harvey and Jenner, Louis, Cooper, Physic and others—to lay his discovery on the altar of universal charity and benevolence.

We can easily see how Dr. Morton was led into the course that he took in regard to patenting his discovery; and from his statement we are bound to believe that the illiberal and unprofessional idea of taking such a step, never originated with him.

Time will show that the course Dr. Morton has pursued, or some other cause—or, perhaps, this in connection *with* others—has given an impulse in regard to this very principle savors of quackery and illiberality in any form. The proceedings of our late annual meeting show to the world, that we are influenced by a spirit like this, and with no prophetic eye, we declare, that at every subsequent meeting, it will continue to influence us, until our Association shall be crowned with that true dignity designed by its original founders.

A liberal profession like ours, devoted to the amelioration of the condition of humanity, a profession which joins hand in hand with the noble one of medicine and surgery, in bestowing happiness and relief to our great brotherhood of the human race, so far *as* regards the *profession*, have no right to *secrets* and *patents ;* and should any of its members become obstinate in regard to their professed "rights," they at once rebel against the *spirit* of our *fraternity*, forfeit all claims to our countenance, and should be regarded accordingly.—*Cazenovia Ed.*

