# Correction

**Published:** 1848-01

**Authors:** 


### Miscellaneous Notices.

"Foul murder has been done—lo! here's the proof!"—*Old Play.*

*Correction.*—"OH for the days of old times of typography, when operatives in the art could render the ancients; when Caxton translated 'Ye Seyge of Troye' from the language of Greece! Would that, in this latter age, when Champollion has deciphered the hieroglyphics of Egypt, when the spirit of inquiry is every where abroad; some one might be found, who could continue to shelter from typical aggression a writer for the press!"

Thus wrote the venerable 'Dr. Ollapod,' while the smarting victim of a proof reader; and thus do we, writhing under the same thorn which called forth the prayer, endorse the apostrophe! We, too, have been victimized; we, too, have been made to endorse sentiments which we never wrote, to call words by wrong names, and to put our signature to unmeaning sentences which scarce bear a semblance to their outraged derivatives in the copy! A "jury of investigation" *might* decide our chirography to be of a character, that it would require another Champollion to decipher it; or they might charge the printer with design, in giving us a voice other than our own! but we will anticipate neither result, and let it be sufficient for us to recount our misfortunes as they are.

We have had the small-pox and the fever and ague, and also a slight touch of the real Asiatic cholera, but we never suffered more than while reading our article for the first time in print, in the October No. of the Journal. If we have an enemy, it would have been greatly to his satisfaction to have been present on the occasion. Had he seen us perspire with rage, while contemplating a distorted sentence, or bite our lips, even to bleeding, with vexation, at seeing the worst possible word in place of the right one, or go into contortions that would do credit to Gabriel Ravell while we discovered some outrageous *"out"* of a line or two in length. If he had not been wholly insatiable, and any ways kind, he would have brushed out the whole score of our offences, and forgiven all of our transgressions!

The first particular spasm we experienced occurred just as our eyes fell upon the word ***resuscitation,*** on the 79th page, in place of ***vesication,*** as we thought we wrote it. "***Blister*** the printer," said we "how fortunate for him that mountains and rivers are between us!" Although vesication is sometimes resorted to to produce resuscitation, yet we apprehend there is a slight difference in their signification—at least were every nervous twinge we experienced during the *reading*, a blow at the printer, he would have presented a *scarcely* living specimen of vesication, and in great need of resuscitation, even at the expense of the villanous ether which *he* advocates for this purpose!

"*Stentorious*" in place of *Stertrous,* on the opposite page, gave us no new feelings of delight, and we really began to blame Dr. Webster that he had not taught *every one* the difference between apoplectic breathing and *thunder!* Yet onward we went, fearing at every step, wading through comma-less sentences of such length that we were obliged to hold our breath as long as a pearl-diver to read them; blaming, forgiving, exonerating the printer, and, finally, anathematizing ourselves and bad writing, until we found ourselves at the bottom of the 85th page; and here *was* an opportunity for charity to have full scope; although we had shouldered everything else, we could not though we would, be of any service here. Here, in horticultural parlance, was a kind of "shortening-in" process that would be an example to a professed gardener—may we produce the better fruit for it the coming season! Here, was actually an "*out*" of a whole line or more, which was certainly in the copy. Had it occurred at any other place in the article, it would have been of less consequence, but here, where we wished to be particularly emphatic, the error comes more than ever, *out of place.* But we will not enlarge upon a subject so disagreeable, and promise the printer that if he will forgive all of our hard thoughts, we will endeavor to write a better hand hereafter.

With this No. we "drop a line" to each of our subscribers. By pasting it at the bottom of the 85th page, and also by drawing a pencil across and correcting the two other errors referred to, they will do us a favor which we promise never to ask again.—***Cazenovia Ed.***

[Our colleague has certainly some cause of complaint, but the deciphering of the manuscript of the article in question was as troublesome to the compositor and proof-reader, as the typographical errors which occurred in the printing has been annoying to him.—***Balt. Ed.***]

